# The mental representations of fractions: adults' same–different judgments

**DOI:** 10.3389/fpsyg.2013.00385

**Published:** 2013-07-01

**Authors:** Florence Gabriel, Denes Szucs, Alain Content

**Affiliations:** ^1^Laboratoire Cognition, Langage and Développement, Faculté des Sciences Psychologiques et de l'Education, Université Libre de BruxellesBrussels, Belgium; ^2^Department of Experimental Psychology, Centre for Neuroscience in Education, University of CambridgeCambridge, UK

**Keywords:** fractions, magnitude processing, symbolic distance effect, numerical cognition, same-different task

## Abstract

Two experiments examined whether the processing of the magnitude of fractions is global or componential. Previously, some authors concluded that adults process the numerators and denominators of fractions separately and do not access the global magnitude of fractions. Conversely, others reported evidence suggesting that the global magnitude of fractions is accessed. We hypothesized that in a fraction matching task, participants automatically extract the magnitude of the components but that the activation of the global magnitude of the whole fraction is only optional or strategic. Participants carried out same/different judgment tasks. Two different tasks were used: a physical matching task and a numerical matching task. Pairs of fractions were presented either simultaneously or sequentially. Results showed that participants only accessed the representation of the global magnitude of fractions in the numerical matching task. The mode of stimulus presentation did not affect the processing of fractions. The present study allows a deeper understanding of the conditions in which the magnitude of fractions is mentally represented by using matching tasks and two different modes of presentation.

## Introduction

Current evidence about how fractions are represented in the human brain is controversial. Numerical abilities are currently thought to rely on an innate biological system representing approximate magnitude (Dehaene, 1997). Gallistel et al. (2005) extended this view by suggesting that the magnitude coding system has properties co-extensive with mathematical real numbers and allows the approximate representation of ratios, a claim supported by evidence from preverbal children (McCrink and Wynn, 2007) as well as primates (Vallentin and Nieder, 2008).

If the mental magnitude system is indeed apt to represent continuous quantities, ratios, and proportions, it would also provide a natural support for representing the magnitude of fractions. However, fractions constitute a major stumbling block in maths education (Mack, 1993), and this difficulty suggests that the link between fraction symbols and their magnitude (i.e., their rational value) is not acquired easily. Hence, a question has recently been raised whether adults process fractions by accessing their global magnitude or only the value of their component numerals.

Bonato et al. (2007) were the first to address this issue by using the symbolic distance effect in the context of fractions. The symbolic distance effect (Moyer and Landauer, 1967) refers to the fact that it is harder to compare close numbers than more distant numbers. Bonato et al. (2007) reasoned that if fractions are compared through their global magnitude, performance should be influenced by the overall distance between fractions (i.e., the numerical distance between the corresponding real numbers) and not by the distance between their components (i.e., the distance between numerators and denominators). Conversely, if fractions are compared componentially, only the distance between numerators and denominators should affect participants' performance. Bonato et al. (2007) observed only componential distance effects and concluded that the magnitude of fractions was not accessed, but later studies reported different findings. Kallai and Tzelgov (2009) found that the magnitude of fractions was not evoked in an intentional comparison of pairs of fractions, but they also showed that when a fraction was compared to a natural number, participants relied first on the magnitude of components and then accessed the global magnitude. Other studies with fraction comparison reported that the global magnitude of fractions can be accessed (Meert et al., 2009, 2010; Schneider and Siegler, 2010). Furthermore, Ischebeck et al. (2009), and Jacob and Nieder (2009) used functional magnetic resonance imaging (fMRI) in comparison and neural adaptation tasks, respectively. The global magnitude of fractions modulated the activity of areas of the intraparietal sulcus in both studies. These findings were taken as evidence that the global magnitude of fractions is mentally represented. In summary, evidence is controversial; there is support for both the componential and global magnitude views. However, the conclusions may be related to the particular strategies that participants can endorse as a function of the characteristics of stimuli and tasks. Faulkenberry and Pierce (2011) have shown that people can represent the overall magnitude of fractions but it depends mainly on the strategy used to compare fractions and on the nature of the fractions being compared.

In this study, we further contrasted the global/componential hypotheses by using a task which has the potential of providing clearer evidence and has not been previously used in this domain. We used a matching task in which participants had to decide whether two fractions were the same or different. Recently, Van Opstal and Verguts (2011) argued that the distance effect in overt number comparison tasks may not be a marker of the magnitude representation but rather emanate from response processes, as a consequence of the strength of stimulus–response mappings. In contrast, matching tasks where participants simply decide whether two numbers are the same or not are directly related to magnitude processing. This conclusion is in line with other theories suggesting that paradigms in which intentional strategies are minimal, such as same/different paradigms, are more appropriate to investigate basic features of mental representations than intentional paradigms (Tzelgov and Ganor-Stern, 2004).

Here we used two versions of the matching task to determine how the magnitude of fractions is processed. In both tasks, there were three types of items: fractions could be identical (same numerator and denominator, e.g., 1/2_1/2); equivalent (same magnitude but different numerators and denominators, e.g., 1/2_2/4); or different (different magnitudes, e.g., 1/2_3/4). In the physical matching task, participants had to answer “Same” if the two fractions presented shared the same physical identity, and “Different” in all other cases. In the numerical matching task, the “Same” response was expected when pairs of fractions shared the same magnitude that is for identical and equivalent fractions. The symbolic distance effect was used to investigate whether participants rely on the magnitude of the components or on the overall magnitude of fractions to make a decision.

The physical and numerical matching tasks probe different levels of automaticity in fraction processing. Magnitude activation is not required to perform the physical matching task. However, it is well known from studies examining integer processing that activation of integer magnitude is involuntary (Henik and Tzelgov, 1982; Dehaene and Akhavein, 1995; Szucs et al., 2007). Hence, even in the physical matching task we expected componential distance effects (distance effects between numerators and denominators). Furthermore, in the physical matching task, equivalent pairs of fractions (1/2 and 2/4) denote two different stimuli, but they represent the same rational number. Thus if the fractional magnitude is activated involuntarily, a difference between equivalent and different pairs should be observed, as the numerical identity of equivalent pairs would interfere with the (negative) physical decision. On the other hand, in the numerical matching task, magnitude activation is required to perform the task. Hence, both componential and global distance effects were expected in this task. We can also predict that if judging physical identity is faster than judging equivalence (even if the last processing is automatic) and if participants rely on physical identity for identical pairs of fractions in the numerical matching task, response times (RTs) will be smaller in the identical condition than in the equivalent condition.

Experiment 2 further assessed the automatic activation of fraction magnitude by presenting the two fractions sequentially. This experiment checked for a potential strategic effect which may affect global/componential processing. The task was the same as in Experiment 1 except that the two to-be-compared fractions were presented one after the other. This experiment was designed along the lines of Zhou et al. (2008) who suggested that two-digit number comparison is componential (i.e., each digit was processed as a decade digit and as a unit digit) when stimuli are presented simultaneously, but global when presented sequentially. Zhou et al. (2008) explained their finding by the fact that participants stored the first number as a whole in short-term memory before processing the second number. In a similar way, we reasoned that a sequential presentation might favor the activation of global magnitude as the sequential task would demand to encode the first fraction into memory and participants would have more time and opportunity to process each fraction. Therefore, we examined whether changing the presentation of fractions from simultaneous to sequential would result in the appearance of a global distance effect in Experiment 2.

## Methods

### Participants

Twenty-seven psychology students from the Université Libre de Bruxelles took part in Experiment 1 and 33 in Experiment 2. Five participants in Experiment 1 and 8 participants in Experiment 2 were excluded because their score was lower than 50% in one of the conditions, leaving a total sample of 22 participants in Experiment 1 (15 women, 23 right-handed) and 25 participants in Experiment 2 (20 women, 24 right-handed). Mean age was 22 years (ranging from 18 to 33 years). They received monetary compensation for their participation. The study was approved by the Research Ethics Committee of the Faculty of psychology and education sciences at the Université Libre de Bruxelles.

### Stimuli

Stimuli were pairs of fractions, divided into three categories: Identical, Equivalent and Different, and included a large range of fractions with denominators from 2 up to 18 and numerators from 1 up to 17. Only proper fractions (i.e., global magnitude < 1) were used. Fractions with denominator 10 were not used because they might easily be transformed into decimal numbers.

Identical fractions shared the same components, that is, same numerators and same denominators (e.g., 1/2_1/2). Equivalent fractions had the same magnitude, but different numerators and denominators (e.g., 1/2_2/4). Different fractions differed in terms of magnitude and components (e.g., 1/2_3/5). In the Different condition, the distances between denominators, numerators, and fraction magnitude were manipulated (see Table 1). Fractions with the same numerator, same denominator, or no common components were used.

**Table 1:** **Mean global and componential distances for pairs of fractions in the different condition**.

	**Physical matching task**		**Numerical matching task**	
	**Mean ± *SD***	**Min-Max**	**Mean ± *SD***	**Min-Max**
Global	0.23 ± 0.15	0.02–0.78	0.22 ± 0.14	0.01–0.78
Numerator	3.8 ± 2.6	0–13	3.6 ± 2.4	0–13
Denominator	4.5 ± 2.9	0–14	4.6 ± 2.9	0–14

In the physical matching task, there were 140 pairs of fractions for which participants had to answer “Same” (i.e., 140 Identical pairs) and 140 pairs of fractions for which they had to answer “Different” (i.e., 70 Different pairs and 70 Equivalent pairs). All possible fractions with numerators going from 1 up to 17 and denominators going from 2 to 18 (excluding 10) were generated, thereby providing 136 Identical fractions. Four of these Identical fractions were repeated in order to get a set of 140 Identical pairs. All 70 possible Equivalent pairs with denominators from 2 to 18 (excluding 10) were used. There were a total of 70 Different pairs of fractions for which componential and global distances were manipulated.

In the numerical matching task, stimuli leading to the answer “Same” were 70 Identical and 70 Equivalent pairs of fractions, and there were140 Different pairs leading to the answer “Different”. We controlled for numerical distances for fractions in the Different condition for both tasks. Numerical distances for pairs of fractions in the Different condition are given in Table 1. Distances were matched between the physical matching task (70 pairs) and the numerical matching task (140 pairs).

A total of 560 trials was presented to each participant, 280 in each task. Stimuli were split into two blocks for each task, preceded by a short training block of 10 pairs of fractions. Training items consisted of fractions with denominators 10, 19, 20, and 40.

In order to control for interrelationships between numerators, denominators and global distance in the Different condition, we ran correlation analyses between global and component distances of fraction pairs. In the physical matching task, none of the correlations reached significance (*r_ng_* = 0.22; *r_dg_* = −0.13; *r_nd_* = 0.22; all *p*s > 0.05). In the numerical matching task, the correlation between the distance between numerators and the global distance was significant, *r_ng_* = 0.34; *p* < 0.001. The correlation between numerator and denominator distances was also significant, *r*_nd_ = 0.29, *p* < 0.001, and so was the correlation between denominator and global distances, *r_dg_* = −0.23, *p* = 0.005.

### Procedure

Presentation and data collection were controlled by Psyscope. Each pair of fractions was presented horizontally, each occupying a square space of 250 pixels. Black characters were presented on white background. Each pair of fractions was composed of different fonts (Arial or Brush) and different vincula (horizontal or oblique bar) in order to impede pure visual matching (see Cohen, 2009). In Experiment 1, a fixation cross appeared on the screen for 300 ms, followed by a 200 ms blank screen. Both fractions then appeared simultaneously for 5000 ms or until the participant gave a response, followed by a 200 ms inter-stimuli interval. The side of the larger fraction was counterbalanced across trials. In Experiment 2, the two fractions were presented sequentially in the center of the screen. The first fraction of a pair appeared for 200 ms, followed by a 200 ms blank screen. Then the second fraction of the pair appeared during 5000 ms or until a response was given. The order of the larger fraction was counterbalanced across trials. RTs were measured from the onset of the second fraction.

Participants were seated in a quiet room, facing a screen and responded by pressing the “m” or the “q” key of an AZERTY keyboard for “Same” and “Different” respectively. They were asked to answer as quickly and accurately as possible. In both tasks, a training block of 10 trials preceded two experimental blocks of 140 trials each. Participants were allowed a short break after each block. The order of the tasks was counterbalanced.

## Results

Trials for which there was an incorrect response were removed prior to RTs analyses. As can be seen in Table 2, accuracy was similar overall in the two experiments, whereas latencies were shorter in Experiment 2. In the physical matching task, accuracy was relatively high for the three types of trials and latencies were shorter for “Same” than for “Different” responses. A different pattern was observed in the numerical matching task, in which equivalent pairs caused many more errors and led to much longer latencies than the other pairs.

**Table 2:** **Mean accuracy (in %) and mean RTs (in ms) for the physical matching task and the numerical matching task in Experiment 1 and Experiment 2**.

	**Physical matching task**			**Numerical matching task**		
	**Identical pairs**	**Equivalent pairs**	**Different pairs**	**Identical pairs**	**Equivalent pairs**	**Different pairs**
**EXPERIMENT 1**						
Accuracy	97.2	98.4	94.9	98.3	70.9	93.3
	[2.0]	[2.0]	[2.7]	[1.6]	[8.6]	[3.3]
RTs	800	838	864	1008	2162	1914
	[116]	[135]	[144]	[163]	[334]	[409]
**EXPERIMENT 2**						
Accuracy	95.4	97.9	96.6	96.6	74.5	92.4
	[5.7]	[2.3]	[3.3]	[5.0]	[9.7]	[5.7]
RTs	666	777	783	799	1860	1522
	[183]	[206]	[184]	[206]	[373]	[339]

Standard deviations are given between brackets.

### Anovas

Accuracy and RTs were analysed by repeated measures analyses of variances (ANOVAs). ANOVAs had a type factor (3 levels: Identical, Equivalent, and Different) and an order factor (2 levels: physical matching/numerical matching and numerical matching/physical matching). These ANOVAs were run in order to investigate a potential automatic processing of the fraction magnitude depending on the condition.

#### Physical matching task

In Experiment 1, accuracy did not differ across conditions, *F* < 1, and there was no significant order effect, *F* < 1. In contrast, RTs differed across conditions: *F*_(4, 26)_ = 6.12, *p* = 0.004, η^2^_*p*_ = 0.59. There was no significant order effect, *F* < 1. *Post-hoc* Tukey tests showed that participants were significantly faster in the Identical condition than in the Different condition (*p* = 0.006) and in the Identical than in the Equivalent condition (*p* = 0.02).

In Experiment 2, accuracy did not differ across conditions, *F*_(2, 32)_ = 1.07, *p* = 0.35, but there was a significant difference between conditions for RTs, *F*_(2, 32)_ = 17.2, *p* < 0.001, η^2^_*p*_ = 0.38. Participants were significantly faster in the Identical condition than in the Different (Tukey *post-hoc*: *p* < 0.001) and Equivalent conditions (Tukey *post-hoc*: *p* < 0.001).

#### Numerical matching task

In Experiment 1, performance was worse for the Equivalent condition than for the Different and Identical conditions, *F*_(2, 52)_ = 110.6, *p* < 0.001, η^2^_*p*_ = 0.86 (Tukey *post-hoc*: Equivalent vs. Different: *p* < 0.001; Equivalent vs. Identical: *p* < 0.001). There was no order effect, *F* < 1. Participants were significantly faster for Identical than for Different and Equivalent fractions, *F*_(2, 52)_ = 233.55, *p* < 0.001, η^2^_*p*_ = 0.95 (Tukey *post-hoc*: Identical vs. Different: *p* < 0.001; Identical vs. Equivalent: *p* < 0.001). There was no significant order effect, *F* < 1.

In Experiment 2, there was a significant difference in accuracy between conditions *F*_(2, 64)_ = 77.47, *p* < 0.001, η^2^_*p*_ = 0.79. Participants had poorer performance for Equivalent fractions than for Different fractions (Tukey *post-hoc*: *p* < 0.001) and Identical fractions (Tukey *post-hoc*: *p* < 0.001). The repeated measures ANOVA on RTs also showed a significant difference between conditions, *F*_(2, 64)_ = 157.8, *p* < 0.001, η^2^_*p*_ = 0.89. RTs were slower for Equivalent fractions than Different and Identical fractions (Tukey *post-hoc*: *p* < 0.001).

#### Comparison across experiments

We ran additional 2 × 2 × 3 ANOVAs (Experiment × Task × Condition) on accuracy and RTs on the common stimuli in the three conditions of each task. For accuracy, we found a significant main effect of Task, *F*_(1, 45)_ = 1.1, *p* < 0.001, η^2^_*p*_ = 0.75. Participants' performance was better for the physical task. There was a significant main effect of Condition, *F*_(2, 90)_= 121, *p* < 0.001, η^2^_*p*_ = 0.72. Performance was significantly worse for Equivalent fractions than for Different and Identical fractions (Tukey: *p* < 0.001). There was also a significant interaction between Task and Condition, *F*_(2, 90)_ = 1.2, *p* < 0.001, η^2^_*p*_ = 0.70. Accuracy for Equivalent fractions was worse than all the other conditions in both tasks (Tukey: *p* < 0.001). For RTs, there was a significant main Experiment effect, *F*_(1, 45)_ = 13.5, *p* < 0.001, η^2^_*p*_ = 0.23. Participants were faster in Experiment 2 when stimuli were presented sequentially. There was also a significant main Task effect, *F*_(1, 45)_ = 4.2, *p* < 0.001, η^2^_*p*_ = 0.90; and a significant main Condition effect, *F*_(2, 90)_ = 4.2, *p* < 0.001, η^2^_*p*_ = 0.90. We also found a significant Task × Condition interaction, *F*_(2, 90)_ = 3.8, *p* < 0.001, η^2^_*p*_ = 0.88. *Post-hoc* tests showed no significant difference between Different and Equivalent fractions in the Physical task (Tukey: *p* = 0.99) and significant differences between all the others conditions and tasks (Tukey: all *p*s < 0.001).

### Regression analyses

Further analyses were run to assess whether componential or global distances affected performance on different pairs of fractions in either task. Accuracy and RTs were analyzed by fitting linear mixed-effect regression models (Baayen et al., 2008) including participants and items as random factors. Given their binomial distribution, a logistic transform was performed for inference tests on the accuracy data (Quené and Van den Bergh, 2008). The significance level of 0.05 was used for all statistical analyses. We compared models including either the global distance between fractions, the distance between numerators and denominators, or the three distances, and reported the statistics for the best fitting model^1^.

#### Physical matching task

In the physical matching task of Experiment 1, the model including all three parameters was a better fit for accuracy, but global distance had an negative influence (fraction distance: *z* = −2.30, *p* < 0.025; denominator distance: *z* = 2.63, *p* < 0.01; numerator distance: *z* = 2.64, *p* < 0.01). The global distance effect was mainly caused by an outlier (i.e., 14/15_2/3). Excluding that observation yielded different statistical results and only the distance between denominators was still significant (fraction distance: *z* = −2.13, *p* = 0.08; denominator distance: *z* = 2.64, *p* = 0.03; numerator distance: *z* = 2.31; *p* = 0.06). In Experiment 2, none of the parameters reached significance.

For RTs, Experiment 1 showed significant facilitation for the distance between numerators and the distance between denominators (*t* = −1.81, *p* < 0.070; *t* = −3.854, *p* < 0.001), whereas no significant effect was observed in Experiment 2. Altogether, the results for the physical matching task indicate contributions of the fraction components, but only in the simultaneous situation.

#### Numerical matching task

In the numerical matching task, results showed an influence of the global magnitude of fractions. For accuracy, the distance between the global magnitude of fractions improved performance both in Experiment 1 (*z* = 2.22, *p* < 0.03) and in Experiment 2 (*z* = 2.73, *p* < 0.007). For RTs, both experiments concurred in producing significant effects of all three variables, with the distance between the global magnitude of fractions reflecting facilitation and both componential distances producing slightly negative effects (Experiment 1: fraction distance: *t* = −2.68, *p* < 0.01; denominator distance: *t* = 2.15, *p* < 0.02; numerator distance: *t* = 2.47, *p* < 0.006; Experiment 2: fraction distance: *t* = −2.09, *p* < 0.01; denominator distance: *t* = 2.63, *p* < 0.002; numerator distance: *t* = 2.49, *p* < 0.006).

## Discussion

We investigated how fractions are processed and represented in adults. We aimed to determine whether fraction magnitude processing was componential or (also) relied on the global fraction magnitude in a new experimental setting. Two tasks were used: a physical and a numerical matching task. Numerical distance effects observed in matching tasks are thought to be directly related to the mental representations of numbers (Van Opstal and Verguts, 2011) and therefore are more appropriate than comparison tasks to assess the processing of numbers. In the physical matching task, participants decided whether two fractions were physically identical. In the numerical matching task, participants decided whether fractions had the same numerical magnitude. Our main finding is that the global magnitude of fraction is not automatically activated, but that it can be activated under specific experimental conditions. Only componential distance effects were found the physical matching task. However, global distance effects were found in the numerical matching task.

In the physical matching task of Experiment 1, participants were faster in the Identical condition than in the Equivalent and Different conditions, suggesting that they relied on perceptual cues and not on the global magnitude of fractions. We first observed a significant negative effect of the global distance for accuracy in different pairs, but this effect disappeared after an outlier was removed from the analyses. Componential distance effects were observed for RTs, but only in the simultaneous condition. In the numerical matching task, participants had poorer performance and were slower in the Equivalent condition than in the Identical and Different conditions. Poor performance in the Equivalent condition suggests that participants often failed to identify the equivalence of fractions. The pattern of results in the numerical matching task (i.e., poorer performance for Equivalent fractions and failure to detect the numerical equivalence when the components differed between fractions) might also suggest that participants tended, at least in part, to rely on physical identity and/or used simplification procedure when they were processing reducible fractions. A limitation of this study is that the key assignment was kept constant. However, the button assignment for Equivalent pairs of fractions changes from one task to the other and we observe a difference in RTs between Equivalent fractions and Different fractions in the numerical matching task but not in the physical matching task. Thus, it is unlikely that there were any confounds linked to the key assignment.

Experiment 2 used the same experimental paradigm as Experiment 1, but the presentation of the stimuli was sequential rather than simultaneous. We expected that the processing of global fraction magnitude would be facilitated by the sequential presentation (Zhou et al., 2008). Our results showed a global distance effect in the numerical matching task. However, there were no significant effects in the physical matching task. Thus, the processing of the global magnitude of fractions was not affected by changing the stimulus presentation mode from simultaneous to sequential. The difference in processing resides in the type of task instead of the presentation of the stimuli.

The present data support previous results reporting global distance effects with fractions in overt number comparison tasks (Ischebeck et al., 2009; Meert et al., 2009, 2010). As suggested by Meert et al. (2010), fraction processing could take place on a continuum from componential to global. In the physical matching task of both experiments, participants only used componential strategies since they could base their judgment on the presence of identical components. They were successful in comparing only the components without accessing the global magnitudes. This is in agreement with the studies of Bonato et al. (2007) and Kallai and Tzelgov (2009).

However, findings in those studies may again be due to the specific task context and cannot be considered a general property of fraction processing. Bonato et al. (2007) used a large amount of fractions with unit numerators (e.g., 1/*x*) in their experiments 1 and 2. Obviously, this type of fraction encourages participants to focus solely on the denominators. Furthermore, in their Experiments 3 and 4, the size of the numerators was always consistent with the global size of the fractions, again, favoring comparison strategies based on separate component comparisons. Kallai and Tzelgov (2009) contrasted numerical and physical comparison of fractions with a numerical Stroop paradigm. Results showed that participants preferred to use strategies based on integer numbers, indicating that there was no unique representation of the global magnitude of fractions in long-term memory. However, again, in their first and fourth experiment, only fractions of the form 1/*x* were used. Therefore, participants were induced to base their judgment on the denominators only. In the second experiment, proper and improper fractions were used, but the larger the numerator was, the larger the global magnitude of the fraction was, encouraging the use of componential strategies. In the third experiment, familiar fractions were used and they observed hybrid strategies in the numerical comparison. This indicates that the nature of the fractions used could have influenced the type of processing.

At the other end of the continuum, the numerical matching task induced a global processing of the magnitudes of fractions. In this condition, exact numerical magnitude comparison of fractions was required and the use of componential strategies was compromised. Moreover, as shown by Meert et al. (2010), various kinds of fractions were presented, and componential strategies were then made less efficient. This is also compatible with fMRI data showing that the global distance effect modulated activation in the right intraparietal sulcus (Ischebeck et al., 2009; Jacob and Nieder, 2009).

To sum up, the global magnitude of fractions can be accessed under specific circumstances. This study was the first to use a matching task with fractions. The numerical distance effect observed in matching task is thought to originate from number representation rather than from a decision process (Van Opstal and Verguts, 2011). Thus, the global distance effect observed in the numerical matching task indicates that the magnitude of fractions can be accessed. However, we did only observed distance effects between the components of fractions in the physical matching task, suggesting that participants relied on componential strategies. Furthermore, we also showed that the mode of presentation of the stimuli did not affect their processing contrary to what has been shown for multi-digit numbers. In conclusion, our results show that the access to the global magnitude representation of fractions is not automatic and is invoked only due to task demands.

### Conflict of interest statement

The authors declare that the research was conducted in the absence of any commercial or financial relationships that could be construed as a potential conflict of interest.

## Footnotes

^1^We examined zero-order and partial correlations between each distance and RTs. As we did not observe any change in the sign nor in the strength of the relation, we could exclude any potential suppression or redundancy effect (see Tzelgov and Henik, 1991).
